# Physician-Targeted Interventions in Antibiotic Prescribing for Urinary Tract Infections in General Practice: A Systematic Review

**DOI:** 10.3390/antibiotics11111560

**Published:** 2022-11-05

**Authors:** Stefan Cox, Kelly Lo-A-Foe, Minke van Hoof, Geert-Jan Dinant, Guy Oudhuis, Paul Savelkoul, Jochen Cals, Eefje de Bont

**Affiliations:** 1 Department of Family Medicine, Maastricht University, P. Debyeplein 1, 6229 HA Maastricht, The Netherlands; 2 Department of Medical Microbiology, Maastricht University Medical Centre+, Universiteitssingel 40, 6229 ER Maastricht, The Netherlands

**Keywords:** systematic review, urinary tract infections, family medicine, antibiotics, antibiotic stewardship

## Abstract

Urinary tract infections (UTIs) are the most common reason for women to consult a general practitioner (GP). While UTIs are self-limiting in half of cases, most women are prescribed antibiotics, often in discordance with established guidelines. Researchers have employed different interventions to improve GPs’ prescribing behavior, especially for respiratory infections, but it is uncertain whether these are effective for UTI care. Therefore, we performed a systematic review, including (cluster) randomized clinical trials investigating the effect of interventions targeted at GPs to improve antibiotic prescriptions for UTI. From September to December 2021 we searched the Medline, Web of Science, and CENTRAL databases, ultimately including ten studies describing eleven trials. We determined the effect of the interventions on the decision to prescribe and on the choice of antibiotic. Results showed that most studies employed multifaceted interventions, most frequently including audit & feedback and/or educational meetings. Seven out of nine trials that recorded first-choice prescriptions saw an increased proportion of first-choice antibiotics in the intervention groups compared to control groups. The employed interventions also caused a decreased proportion of at least one broad-spectrum antibiotic in five out of six studies that measured broad-spectrum antibiotic prescriptions. However, the total number of antibiotic prescriptions for UTIs increased in four out of eight studies. Therefore, while effective at influencing GPs’ prescribing behavior, future interventions should also focus on improving the decision to prescribe at all.

## 1. Introduction

Urinary tract infections (UTIs) are the most common reason for women to consult their general practitioner (GP) [1]. Despite the self-limiting course of UTIs in almost 50% of women without risk factors, GPs prescribe antibiotics to three-quarters of the women presenting with urinary symptoms [2,3,4]. Furthermore, it is suggested that two-thirds of the antibiotic prescriptions issued to women presenting with urinary symptoms are inappropriate, i.e., not compliant with the established guidelines [5].

The overuse of broad-spectrum antibiotics can lead to antibiotic resistance in the targeted pathogens. For example, quinolones are some of the more widely used classes of broad-spectrum antibiotics. Quinolones are effective against both gram-positive and gram-negative bacteria and have long since been used for the treatment of UTIs [6,7,8,9]. They act on bacterial type II isomerases, hereby causing bacterial enzymes to fragment the bacterium’s DNA [7]. The method through which quinolones exert their function is dependent on the presence of a serine and acidic residue(s) associated with a water-metal ion bridge within the target enzymes. A point mutation at any of these locations in the target proteins grants the bacterium resistance to quinolone activity, which means that the threshold for bacteria to become resistant to quinolones is low. For example, over 90% of all quinolone-resistant strains have an alteration at the serine site [9]. Overusing quinolones will put evolutionary pressure on wild-type bacteria, which results in the mutant strains becoming predominant. These mutant strains are much more difficult to treat and therefore caution is necessary when employing broad-spectrum antibiotics and quinolones in particular.

Underlying reasons for inappropriate prescriptions are likely multifactorial; qualitative studies show that GPs are often unfamiliar with current guidelines, have misconceptions about the management of UTIs, experience time constraints (leading to “better-safe-than-sorry” prescriptions), and perceive pressure from patients to prescribe as reasons to deviate from the guidelines [10,11,12,13,14].

Deviation from established guidelines leads to overuse and misuse of antibiotics, facilitating the rise of antimicrobial resistance, a key issue in health care globally [15,16]. Because of this, different interventions have been developed in an effort to reduce inappropriate prescribing in outpatient care. Previous reviews have shown that implementation of these interventions for common (mostly respiratory) infections leads to a decrease in inappropriate prescribing of antibiotics in outpatient care [17,18].

However, few studies have investigated interventions aimed at improving UTI care [17,18]. Therefore, we systematically reviewed the literature for physician-targeted interventions aimed at improving GP antibiotic prescribing for UTIs.

## 2. Results

### 2.1. Study Selection

**Figure 1:**
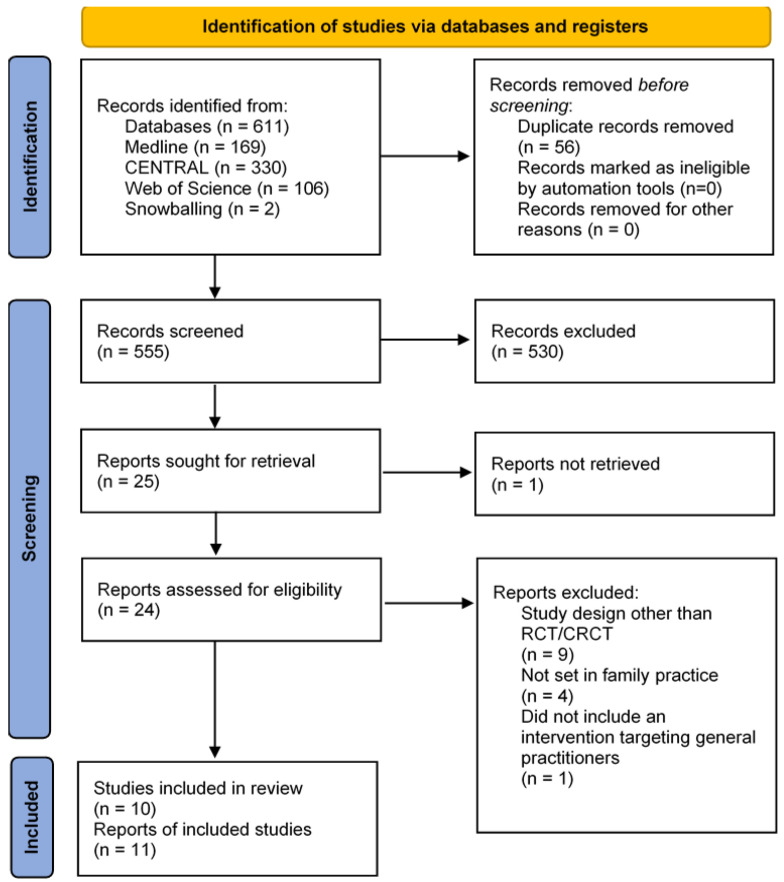
Flow chart of study selection. After examining titles, abstract, and full texts of the studies found during literature search, eight studies were eligible for review. The most common reason for exclusion was the study not being an RCT or CRCT, followed by not being set in family practice and not including an intervention targeted at GPs. Two additional studies were included using the snowballing method. CRCT = cluster randomized controlled trial, RCT = randomized controlled trial, UTI = urinary tract infection.

The literature search yielded 611 results. After removal of duplicates (*n* = 56) and screening of titles and abstracts (*n* = 530), we excluded 586 studies in total. After assessing the full-text versions of the remaining 25 studies, ten articles remained that met the inclusion criteria. Vellinga et al. investigated the effect of two different interventions on GPs’ prescribing behavior in separate trial arms, therefore both arms were viewed as distinct trials for the purpose of this review. Thus, this review includes eleven trials in total (Figure 1).

### 2.2. Study Characteristics

All included studies were cluster randomized controlled trials except for one, which was an individually randomized controlled trial. In total, the studies included over 900 GPs from over 500 practices situated in Europe and Canada. Study details are described in Table 1. Most included trials (*n* = 10) implemented a multifaceted intervention to influence GPs’ prescribing behavior. Eight trials included educational interventions to increase awareness of the current treatment guidelines. Audit and feedback (*n* = 8), educational meetings (*n* = 7), and educational materials (*n* = 5) were interventions that were most frequently employed. Five trials compared the effect of the employed intervention(s) to similar intervention(s) targeting another condition. These comparative conditions were mostly respiratory diseases (*n* = 4). However, Martens et al. compared the effect of the intervention on antibiotic prescriptions to the effect of the intervention on prescriptions for cholesterol-lowering drugs. Trimethoprim, either on its own or in combination with sulfamethoxazole, was the most commonly cited first-choice drug for UTIs (*n* = 7), followed by nitrofurantoin (*n* = 6).

### 2.3. Study Quality

**Figure 2:**
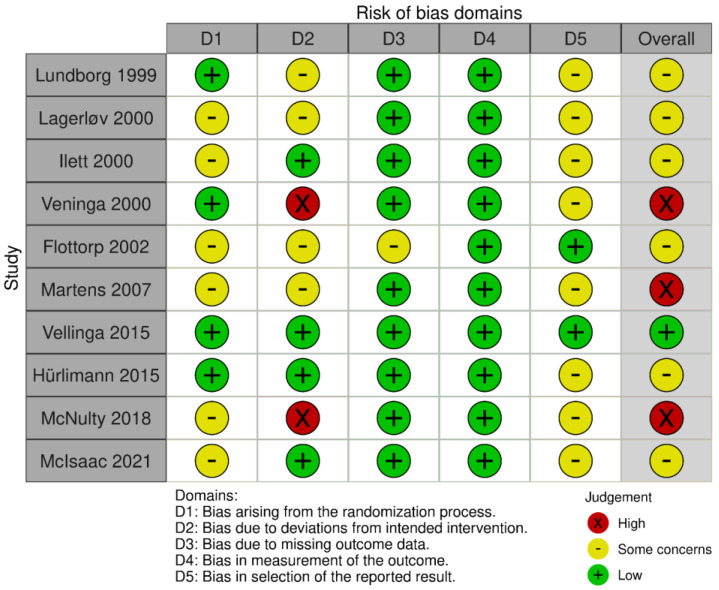
Risk of bias analysis of the included studies. Only one study was judged to have low risk of bias for all domains. We had some concerns about bias in six of the included studies, which mainly arose from the randomization process or the absence of a pre-specified analysis plan. We considered three studies to be of high risk of bias, predominantly because not all clusters assigned to the intervention group received the intervention in these studies [30].

We used the “revised Cochrane risk-of-bias tool for randomized trials (RoB 2)” and the “revised Cochrane risk-of-bias tool for cluster-randomized trials (RoB 2 CRT)” to assess the quality of the included studies [29]. Figure 2 provides an overview of the results of the assessments. We considered only one study to have a low chance of bias. Most of the studies merely stated that randomization had been performed, but not in which way. Therefore, almost all studies were deemed to be at some risk of bias for domain 1: bias arising from the randomization process. Additionally, almost none of the studies reported a pre-specified analysis plan, resulting in concerns of bias for domain 5: bias in selection of the reported result. A high risk of bias was determined to be present in three studies, mainly because these studies contained a substantial amount of participants that did not complete the intervention, despite allocation to the intervention group. Despite the higher chance of bias in these studies, they were still included in further analysis to provide a complete overview of the available literature.

### 2.4. Primary Outcomes

#### 2.4.1. Effect of the Interventions on the Number of Antibiotic Prescriptions

Eight of the included trials reported on the effect that their intervention had on the number of antibiotic prescriptions issued for UTIs. These are listed in Table 2 [19,21,23,25,26,27,28]. Lundborg et al. were the only ones to report a reduction of prescribed antibiotics for UTIs compared to the control group. Four studies reported a significant increase of antibiotic prescriptions for UTIs in the intervention group, while three other studies reported no significant difference between the groups.

#### 2.4.2. Effect of the Interventions on the Appropriateness of Antibiotic Prescriptions

Nine studies measured the effect of their intervention on the appropriateness of the antibiotics prescribed for UTIs and are listed in Table 3 [19,21,22,24,25,26,27,28]. Seven of these nine studies reported a significantly higher proportion of first-choice antibiotics in the intervention group compared to the control group. Even though Martens et al. reported no absolute prescription numbers, they did report an increased proportion of first choice antibiotic prescriptions in the intervention group; 73% (95% CI = 69–80), compared to 57% (95% CI = 52–63) in the control group. However, because no absolute numbers are reported (and data were unavailable after request), we were unable to calculate an odds ratio. McIsaac et al. and McNulty et al. were the only ones to report no significant effect, but they did show a trend towards increased first-choice prescriptions in the intervention group.

#### 2.4.3. Effect of the Interventions on Broad-Spectrum Antibiotic Prescriptions

Six of the included trials reported on the effect of their intervention on broad-spectrum antibiotic use for UTIs and are listed in Table 4 [19,21,25,26,27]. Most studies investigated the effect of their intervention on quinolone and/or penicillin prescriptions. Five trials reported a significantly lower proportion of broad-spectrum antibiotics in the intervention group compared to the control group. McNulty et al. reported no significant reduction of broad-spectrum antibiotic prescriptions for UTIs. Hürlimann et al. saw an increased proportion of penicillin prescriptions in the intervention group concurrent with a decreased proportion of quinolone prescriptions compared to the control group. Martens et al. also investigated the effect of their intervention on quinolone prescriptions for UTIs, but reported the outcome as volumes of quinolone prescribed per GP per 1000 enlisted patients (prescribing rate). However, due to missing absolute numbers, this could not be included in the table. Nonetheless, they report a quinolone prescribing rate of 1.5 (95% CI = 0.8–2.2) in the intervention group compared to 4.6 (2.8–8.1) in the control group [24].

### 2.5. Additional Outcomes

Lagerløv et al. were the only ones not to report on any of the above outcomes. However, Lagerløv et al. did report that their intervention led to a 13.1% relative increase in the proportion of prescribed short antibiotic courses for UTIs (≤4 days) compared to controls, as well as a 9.6% relative decrease in prescribed long antibiotic courses for UTIs (>4 days) [20]. Veninga et al. found a similar effect of their intervention on treatment duration: the average duration of treatments with first-choice drugs decreased from 6.07 defined daily doses (DDD) per prescription to 4.29 DDD per prescription, while treatment duration in the control group did not change significantly (from 5.40 DDD per prescription to 5.51 DDD per prescription) [22]. However, Lundborg et al. reported no significant change in mean treatment duration in both the intervention (7.51 DDD to 7.41 DDD) and control (7.60 DDD to 7.44 DDD) groups [19]. Similarly, McIsaac et al. reported an insignificant difference of number of treatments longer than seven days between intervention (3/185) and control (4/287) groups [28].

In addition to collecting prescribing data in the period the intervention was implemented, Vellinga et al. continued to do so for five months after implementation had ended. They reported no significant change in prescriptions for nitrofurantoin (as a percentage of total antibiotics prescribed) between the intervention period and the five-month follow-up period in the intervention group (63.8% and 57.3%, respectively), while the control group did prescribe significantly more nitrofurantoin after the intervention period (35% and 47.8%, respectively) [25].

## 3. Discussion

### 3.1. Summary

Results of this systematic review show that most physician-targeted interventions on antibiotic prescribing behavior of general practitioners for urinary tract infections were multifaceted, most frequently incorporating interventions to increase awareness about current guidelines around UTIs, as well as prescribing feedback. Almost all trials reported increased prescribing of first-choice antibiotics in the intervention group compared to the control group. This increase in first-choice antibiotic prescriptions was often paired with decreased prescriptions of broad-spectrum antibiotics. However, half of the trials that measured total antibiotic prescriptions for UTIs reported an increased prescription rate in the intervention group compared to the control group.

### 3.2. Explanation of Results

Interventions targeting GPs’ antibiotic prescribing behavior are able to influence GPs’ choice of antibiotic, increasing the odds of first-choice antibiotic prescriptions in seven out of nine trials. McIsaac et al. and McNulty et al. were the only ones to not report a significantly increased proportion of first-choice antibiotic prescriptions in the intervention group compared to the control group, despite the use of interventions that require active participation by GPs, which have been shown to be of greater effect than passive interventions [31,32]. For McIsaac et al., this is likely caused by the comparatively small sample size in this study, since a total of six practices were included accounting for 460 antibiotic prescriptions. These numbers are considerably smaller than for the other included studies. When considering the differences in percentage of first-choice antibiotic use between the groups, we consider it likely that if a larger sample size would have been used, a significant difference would have been found here as well.

McNulty et al. were the only ones with a large sample size to not see an increased proportion of first-choice antibiotics in the intervention practices. This could be due to the intention-to-treat (ITT) analysis that we employed to determine the effectiveness of McNulty et al.’s study, since over half of practices assigned to the intervention arm did not receive the intervention. However, in the original paper the researchers performed a per protocol analysis in addition to the ITT analysis we included, and there they did not see an increase of first-choice antibiotics or a decrease in broad-spectrum antibiotics either. However, they reported a significant decrease of total antibiotics prescribed in intervention practices compared to control practices. Furthermore, the absence of the intended effect might be explained by the way the odds ratios were calculated. McNulty et al. calculated odds ratios using UTI prescriptions as a proportion of total antibiotic prescriptions. However, to measure an increased proportion of first-choice UTI antibiotics, one could also calculate prescription odds by measuring nitrofurantoin prescriptions over all UTI prescriptions, which they define as trimethoprim, nitrofurantoin, and pivmecillinam prescriptions. If we do this, with the added caveat of missing cluster data, we see a significant increased proportion of first-choice antibiotics in the intervention group. Therefore, caution is needed when interpreting the results of this review, since not all outcomes might be as comparable as we would like.

While the different interventions were able to positively influence the choice of antibiotic, we did see an increase of total antibiotics prescribed for UTIs in the intervention group compared to the control group in four out of eight trials. An explanation could be that most interventions emphasized prescribing the correct type of antibiotic, while little to no attention was given to the decision to prescribe at all. The inclusion of recommendations to employ delayed (or back-up) prescriptions in the interventions could also have been a reason for the increased number of prescriptions, since they might lower GPs’ threshold to prescribe. Delayed prescriptions were recommended in the intervention of McIsaac et al. and in one intervention arm of Vellinga et al. However, McIsaac et al. reported no significant increase in total antibiotic prescriptions in the intervention arm. Furthermore, the other intervention arm of Vellinga et al.’s study, which did not include recommendations for delayed prescribing, saw a similar increase in total prescriptions as in the ‘delayed prescriptions’ arm. The other included studies did not mention delayed prescriptions in their interventions or in the included guidelines. It is therefore unlikely that delayed prescriptions are the cause of the increase of total antibiotic prescriptions in these studies. For Flottorp et al., this increase in total antibiotic prescriptions is likely also caused by a difference that was already present at baseline. In addition to prescription data after implementing the intervention, they also recorded prescription data during the eighteen weeks before the implementation. The odds ratio for an antibiotic prescription being prescribed for a UTI was 1.14 (95% CI = 1.02–1.26) at baseline, which is similar to the odds ratio found after the intervention period.

Ilett el al reported the total number of prescriptions for every antibiotic, but did not link the prescriptions to their corresponding infection. Therefore, we considered the prescriptions of trimethoprim and cephalexin to be ‘UTI prescriptions’, since these are the first and second choice antibiotics as indicated in the intervention Ilett et al. employed. However, cephalexin is also indicated for infections other than those of the urinary tract (such as acute pharyngotonsillitis, community-acquired pneumonia, and skin infections), which could cause an overestimation of the number of cephalexin prescriptions for UTIs in this study.

The increase in first choice antibiotics prescribed for UTIs is often paired with a decrease in prescriptions of broad-spectrum antibiotics. This is unsurprising, since in order to increase the proportion of first choice antibiotics, the proportion of other antibiotics has to decrease. Almost every trial that reported on the prescription of broad-spectrum antibiotics for UTIs saw a decreased proportion in at least one of the broad-spectrum antibiotics, mostly quinolones or amoxicillin. McNulty et al. were the only ones to report no change in broad-spectrum antibiotic prescriptions for UTIs, which might be due to similar reasons as described before.

Interestingly, Hürlimann et al. found an increase in the proportion of penicillin prescribed for UTIs in the intervention group. We found that until shortly before the time of study, the guidelines in Switzerland indicated ciprofloxacin as empiric therapy, with amoxicillin or co-trimoxazole as specific therapy. The intervention used in the study did not indicate amoxicillin for the treatment of UTIs, and instead opted for co-trimoxazole as first-choice treatment, with ciprofloxacin and norfloxacin as alternatives. Therefore, less penicillin could be expected to be prescribed for UTIs in the intervention group, as the intervention should convince GPs to prescribe co-trimoxazole instead of amoxicillin. However, the intervention did indicate co-amoxiclav for the treatment of UTIs in pregnant women. Since the absolute number of penicillin prescriptions is fairly low, the difference could be explained by a larger number of pregnant women in the intervention group. However, no data are available regarding the number of complicated or uncomplicated UTIs in each group, so whether this is the cause of the increase remains uncertain. Furthermore, part of the intervention in this study was the recommendation to prescribe penicillin for the treatment of RTIs, which could have had an effect on GPs’ choice of antibiotics for the treatment of UTIs.

Our results are partially in line with previous research findings, indicating that interventions in primary care can lead to improved antibiotic prescribing [17,18]. Previous studies have shown that interventions that require active participation by physicians have a greater effect on their prescribing behavior than passive interventions [31,32,33]. Martens et al. and Hürlimann et al. employed passive interventions (computer reminders and guideline provision combined with prescription feedback, respectively), but still saw an increase of first-choice antibiotics prescribed for UTIs, as well as a decrease in quinolone prescriptions. Computer reminders, as implemented by Martens et al., have been shown to be able to change prescribing behavior in the past [34]. For Hürlimann et al., this might also be caused by the fact that the study was performed in a population of GPs that were members of a sentinel network, therefore the participating physicians were probably more receptive to the intervention and more willing to adapt their prescribing behavior.

### 3.3. Strengths and Limitations

An important strength of this review is that we applied a broad search strategy. Moreover, we chose to only include RCTs in our review to ensure a high level of evidence quality. Study quality of the included studies varied from poor to good. We had some concerns about the methodologic quality of most studies, which was mainly related to selection bias due to the absence of a pre-specified analysis plan. Moreover, participants or personnel were unblinded in nearly all studies. Since most of the included studies were cluster randomized controlled trials, it was nearly impossible for participants or personnel to be unaware of the assigned intervention. Although it was not reported whether this caused deviations from intended interventions, we do not consider this likely given the nature of the interventions, unless this was specified in the studies’ reports.

Our systematic review has certain limitations. First of all, we were unable to perform a meta-analysis due to the heterogeneity in study populations and interventions. This is a problem often faced when analyzing intervention studies that target prescribing behavior [13]. Some trials assessed dispensing data collected from pharmacies or centralized databases, while others collected physicians’ prescription data. An argument in favor of the former would be that changing GPs’ prescribing behavior is futile if it leaves patients dissatisfied, resulting in them consulting a different GP to obtain an antibiotic prescription. In that case, the employed intervention(s) could work wonders in decreasing inappropriate prescribing by GPs, but there would be little to no change in antibiotics consumed. However, since these interventions intended to alter GPs’ prescribing behavior, we feel it would be most sensible to determine the change in the number of prescriptions issued and not necessarily the change in prescriptions filled. The way one could go about this is by determining the number of consultations GPs receive for urinary symptoms over a year, if an antibiotic prescription is provided during these consultations, and if so, which antibiotic is prescribed. This way, changes in total number of prescriptions and changes in the choice of prescriptions for UTIs can be determined the most directly. While the ultimate goal of the physician-targeted interventions is to decrease (broad-spectrum) antibiotic consumption for UTIs, their immediate effect is on the prescribing behavior of the targeted physicians and researchers should look for the effect exactly there. Furthermore, if any intervention(s) is/are proven to be sufficiently effective (and cost-effective), implementation of the intervention(s) nationwide would mean that patients would no longer have the option to consult a GP that has not been subjected to the intervention. This would mean that it would be much more difficult for patients to obtain an antibiotic prescription, unless they show sufficiently severe symptoms or belong to a risk group.

Despite the heterogeneity of data, we recalculated outcomes of interest as ORs to enable comparison and interpretation of the data. A caveat here is that the ORs we calculated are unadjusted for the clusters the GPs were assigned to, since those data were unavailable to us. The effect we calculated might therefore be an overestimation of the actual effect. Still, when only taking into account the trials that provide an adjusted OR, we see at least a trend towards better prescribing in all studies.

While most included studies propose trimethoprim as the first-choice antibiotic for UTIs, perspectives around antibiotic treatment for UTIs have changed in recent years. This is mainly caused by the decreased sensitivity rate of *E. coli* for trimethoprim. In the Netherlands, 12–28% of *E. coli* strains isolated from urine in 2014 were resistant to trimethoprim [35]. In accordance with international guidelines, current Dutch guidelines around UTI care consequently recommended trimethoprim as the third drug of choice, behind nitrofurantoin and fosfomycin [36,37]. Therefore, while trimethoprim was a useful drug to promote at the time of the studies included in this review (and is definitely preferable to quinolones for the treatment of an uncomplicated UTI), any future interventions should focus on the use of any of the other antibiotics recommended by the guidelines.

In this review, we did not examine the feasibility of the different interventions for implementation, at least not in the financial sense. Since we determined the effectiveness of the interventions based on intention-to-treat analyses, any failure to adhere to the intervention (for whatever reason) would have influenced the results. One could argue that this is at odds with our reasoning in the above section, where we state that we were interested purely in the effect of the intervention on prescribing data. If we would apply that same logic here, a per-protocol analysis might have been more prudent, since that would give us the clearest indication of the effectiveness of being subjected to any intervention. However, since we were interested in the effects of the intervention from the perspective of the GP, we felt it was necessary to include analyses that took into account whether the physicians were willing to subject themselves to the interventions. After all, if GPs are unwilling to follow courses or attend workshops, no matter how effective they are, they will be of little success.

### 3.4. Implications for Practice

In a time where the available antibiotics are threatened by antimicrobial resistance, there is an urgent need for improvement in the use of antibiotics. Our findings show promise of some physician-targeted interventions in improving antibiotic prescriptions for UTIs, namely educational outreach and multifaceted interventions with interactive components. Important factors in effectiveness seem to be peer discussion and the use of clear information applicable to the participant’s own experience. Overall, there needs to be more uniformity in outcome measurements to allow adequate comparison between studies. Interventions should also focus on improving the decision to prescribe at all in the case of a UTI, since current interventions have the unintended side effect of increasing the total number of antibiotic prescriptions for UTI, even if they are effective at improving the choice of antibiotic. Additionally, future research needs to take cost-effectiveness of interventions into account to allow accurate estimates of scalability, i.e., whether interventions can be implemented nationwide. Finally, follow-up periods need to be longer so that long-term effects can be assessed, and the sustainability of the intervention can be judged adequately.

## 4. Materials and Methods

### 4.1. Search Strategy

We performed a structured literature search from September through December 2021 in Pubmed, Conchrane Central Register of Controlled Trials (CENTRAL), and Web of Science. The complete search strategy is available in Appendix A. We identified additional studies by scanning the bibliographies of the initially included studies.

### 4.2. Inclusion & Exclusion Criteria

We included randomized controlled trials that studied the effects of physician-targeted interventions on antibiotic prescribing for UTIs in primary care. We excluded studies that did not report data for UTIs or UTI-specific antibiotics, as well as interventions aimed at GPs in nursing homes. Additionally, we only considered studies performed in countries with where GPs have a comparable gatekeeping function. We put these limitations in place to ensure uniformity of patient populations between studies from different countries. We used no language restrictions.

Furthermore, we included studies that used standard procedures as comparator, as well as studies that used interventions for improving prescriptions for non-infectious diseases as comparator. Lastly, we included studies that reported at least one of fourteen interventions from a pre-defined list, which we based on a previously developed taxonomy from the Cochrane Collaboration Effective Practice and Organization of Care (EPOC) (Appendix B).

### 4.3. Study Selection

Three reviewers (KL, MvH, and SC) independently screened retrieved titles and abstracts for full-text assessment. Subsequently, the same three reviewers independently assessed full-text articles of the selected studies for inclusion. Disagreement was resolved by discussion and reaching consensus with a fourth reviewer (EdB).

### 4.4. Quality Assessment

Three reviewers also assessed the methodological quality of the included study independently, using the Cochrane risk of bias 2.0 tool [29]. Discussion with a fourth reviewer resolved any disagreements. We considered studies as either having a “low risk of bias”, a “high risk of bias”, or that there were “some concerns” about the risk of bias.

### 4.5. Data Analysis

Three independent reviewers (KL, MvH, and SC) extracted study characteristics and data using a standardized electronic form. Discussion resolved any differences between the reviewers. In case of missing methodological information, we contacted the corresponding author for inquiry [20,22,24].

The effect of physician-targeted interventions on GPs’ prescribing behavior can be measured in a variety of ways. Therefore, we defined three outcomes as measures of improvement before we started data extraction. These were the proportion of antibiotic prescriptions issued for UTI, the proportion of first-choice antibiotics, and the proportion of broad-spectrum antibiotics (i.e., fluoroquinolones). If odds ratios (ORs) were not already available, we recalculated reported outcomes as ORs with the appropriate 95% confidence intervals (95%CI). We narratively described other outcome measures when considered relevant.

### 4.6. Reporting of Results

Report of our study in this manuscript was performed according to the Preferred Reporting Items for Systematic Reviews and Meta-Analyses (PRISMA) guidelines [38]. The PRISMA checklists for both the abstract and the manuscript proper are available the supplementary material.

## Figures and Tables

**Table 1:** Study characteristics.

Author (Year)	Design	Country	Population	Intervention	Comparator	Primary Outcome(S)	Disease/Drug Target	First Choice Antibiotic
Lundborg (1999) [19]	CRCT	Sweden	GP groups (*n* = 36)	audit & feedback, educational meetings	Multifaceted intervention for asthma	Number of antibiotic prescriptions for UTIs, number of prescriptions for asthma	UTI, asthma	trimethoprim, pivmecillinam, nitrofurantoin
Lagerløv (2000) [20]	CRCT	Norway	GP groups (*n* = 32)	audit & feedback, educational meetings, local consensus processes	Multifaceted intervention for asthma	The difference in proportions of short and long treatments for UTIs and asthma before and after the intervention	UTI, asthma	NR
Ilett (2000) [21]	RCT	Australia	GPs (*n* = 112)	educational materials, academic detailing	Standard care	Number of antibiotic prescriptions for upper and lower RTI and UTIs	UTI, RTI	trimethoprim
Veninga (2000) [22]	CRCT	The Netherlands	GP groups (*n* = 84)	audit & feedback, educational materials, educational meetings	Intervention for increasing corticosteroids prescribing for asthma	The proportion of first-choice drugs dispensed of all prescribed UTIs drugs, the average duration of treatment for first- choice UTI drugs.	UTI, asthma	trimethoprim, nitrofurantoin, sulfamethizol
Flottorp (2002) [23]	CRCT	Norway	GP practices (*n* = 142)	educational materials, educational meetings, patient-mediated interventions, reminders	Multifaceted intervention for sore throat.	Change in antibiotic prescription rate	UTI, sore throat	NR
Martens (2007) [24]	CRCT	The Netherlands	GP practices (*n* = 23)	reminders	Computer reminder system for cholesterol lowering drugs	Prescription according to the guideline recommendation as a percentage of total prescriptions	Bacterial infections, asthma, COPD, high cholesterol	trimethoprim, nitrofurantoin
Vellinga A (2015) [25]	CRCT	Ireland	GP practices (*n* = 30)	audit & feedback, educational meetings, patient-mediated interventions, reminders	Standard care	Proportion of prescriptions for recommended first-line antimicrobials.	UTI	trimethoprim, nitrofurantoin
Vellinga B (2015) [25]	CRCT	Ireland	GP practices (*n* = 30)	audit & feedback, educational meetings, patient-mediated interventions, reminders	Standard care	Proportion of prescriptions for recommended first-line antimicrobials	UTI	trimethoprim, nitrofurantoin
Hürlimann (2015) [26]	CRCT	Switzerland	GP practices (*n* = 140)	audit & feedback, educational materials	Standard care	Percentage of co-trimoxazole prescriptions for UTI, percentage of penicillin prescriptions for RTI	UTI, RTI, COPD	trimethoprim/ sulfamethoxazole
McNulty (2018) [27]	CRCT	UK	GP practices (*n* = 150)	audit & feedback, educational materials, educational meetings, patient-mediated interventions	Standard care	Total oral antibiotics dispensed (per 1000 practice patients, excluding anti-tuberculosis and minocycline)	UTI, RTI	nitrofurantoin
McIsaac (2021) [28]	CRCT	Canada	Primary care clinics (*n* = 6)	audit & feedback, educational materials, academic detailing, patient-mediated interventions	Standard care	Total antibiotic prescriptions for URI, sore throat presentations, acute sinusitis, acute bronchitis, and acute uncomplicated cystitis	Uncomplicated cystitis, acute sinusitis, URI, sore throat, acute bronchitis	NR

COPD = chronic obstructive pulmonary disease, CRCT = cluster randomized controlled trial, GP = general practitioner, NR = not reported, RCT = randomized controlled trial, RTI = respiratory tract infection, URI = upper respiratory infection, UTI = urinary tract infection.

**Table 2:** Intervention effect on total antibiotic prescriptions for UTI.

Study	Intervention (%)	Control (%)	OR	95% CI
Lundborg	1857/205836 (0.9) ^1^	1880/191673 (1.0)	*0.92*	0.86–0.98
Ilett	503/7262 (6.9) ^2^	546/9654 (5.7)	*1.19*	1.17–1.21
Flottorp	1167/2522 (46.3) ^3^	1285/2961 (43.4)	*1.12*	1.01–1.25
Vellinga A	584/743 (78.6) ^3^	521/783 (66.5)	*1.85*	1.47–2.32
Vellinga B	559/738 (75.8) ^3^	521/783 (66.5)	*1.57*	1.25–1.97
Hürlimann	3217/15625 (20.6) ^2^	2744/13327 (20.6)	1.00	0.94–1.06
McNulty	67850/343892 (19.7) ^2^	67210/372427 (18.0)	0.99 *	0.95–1.03
McIsaac	128/161 (79.5) ^3^	92/119 (77.3)	1.12 *	0.71–1.79

^1^ Antibiotic prescriptions for UTI/total prescriptions. ^2^ Prescriptions for UTI/total antibiotic prescriptions. ^3^ Prescriptions for UTI/total UTI consultations. * Adjusted OR and 95% CI as reported by the respective article. Cursive ORs have a *p* < 0.05. OR = odds ratio, 95% CI = 95% confidence interval.

**Table 3:** Intervention effect on first-choice antibiotic prescriptions for UTI.

Study	Intervention (%)	Control (%)	OR	95% CI
Lundborg	1227/1857 (66.1) ^1^	1018/1880 (54.1)	*1.65*	1.37–1.98
Ilett	261/7262 (3.6) ^2^	291/9654 (3.0)	*1.20*	1.01–1.42
Veninga	2456/2760 (89.0) ^1^	2412/2838 (85.0)	*1.43*	1.22–1.67
Martens	NR (73)	NR (57)		*p* < 0.05
Vellinga A	507/743 (68.2) ^3^	345/783 (44.1)	2.7 *	1.8–4.1
Vellinga B	491/738 (66.5) ^3^	345/783 (44.1)	2.0 *	1.3–3.0
Hürlimann	1129/3217 (35.1) ^1^	516/2744 (18.8)	2.16 *	1.19–3.91
McNulty	24394/343892 (7.1) ^2^	23164/372427 (6.2)	1.07 *	1.00–1.15
McIsaac	238/258 (92.2) ^3^	197/232 (84.9)	1.41 *	0.66–3.01

^1^ First-choice antibiotic prescriptions for UTI/total antibiotic prescriptions for UTI. ^2^ First-choice antibiotic prescriptions for UTI/total antibiotic prescriptions. ^3^ First-choice antibiotic for UTI, sore throat, and sinusitis/total prescriptions for UTI, sore throat, and sinusitis. * Adjusted OR and 95% CI as reported by the respective article. Cursive ORs have a *p* < 0.05. CI = confidence interval, NR = not reported, OR = odds ratio.

**Table 4:** Intervention effect on broad-spectrum antibiotic prescriptions for UTI.

Study	Antibiotic	Intervention (%) ^1^	Control (%) ^1^	OR	95% CI
Lundborg	Quinolones	590/1857 (31.8)	829/1880 (44.1)	*0.59*	0.52–0.67
Ilett	Amoxicillin	1447/7262 (19.9)	2151/9654 (22.3)	*0.87*	0.81–0.94
	Amoxicillin + CA	249/7262 (3.4)	333/9654 (3.4)	0.99	0.84–1.17
Vellinga A	Amoxicillin + CA	37/743 (4.9)	81/783 (10.3)	0.4 *	0.3–0.7
	Quinolones	19/743 (2.6)	52/783 (6.6)	0.6 *	0.3–1.00
Vellinga B	Amoxicillin + CA	24/743 (3.2)	81/783 (10.3)	0.3 *	0.2–0.6
	Quinolones	16/738 (2.2)	52/783 (6.6)	0.7 *	0.3–1.4
Hürlimann	Penicillins	161/3217 (5.0)	99/2744 (3.6)	*1.41*	1.09–1.82
	Quinolones	1174/3217 (36.5)	1430/2744 (52.1)	*0.53*	0.48–0.59
McNulty	All	42837/343892 (12.5)	49735/372427 (13.4)	0.99 *	0.93–1.05
	Amoxicillin + CA	24017/343892 (7.0)	28784/372427 (7.7)	0.97 *	0.89–1.05
	Cephalosporins	8551/343892 (2.5)	10727/372472 (2.9)	1.00 *	0.87–1.16
	Quinolones	10269/343892 (3.0)	10244/372427 (2.8)	1.04 *	0.95–1.14

^1^ Specific antibiotic prescriptions for UTI/total antibiotic prescriptions for UTI. * Adjusted OR and 95% CI as reported by the respective article. Cursive ORs have a *p* < 0.05. CA = clavulanic acid, CI = confidence interval, OR = odds ratio, NR = not reported.

## Data Availability

Data are available upon reasonable request.

## Supplementary Materials

The following supporting information can be downloaded at: https://www.mdpi.com/article/10.3390/antibiotics11111560/s1, File S1: PRISMA_2020_checklist Antibiotics.pdf. File S2: PRISMA_2020_abstract_checklist antibiotics.pdf.

## Registration and Protocol

This review was not registered, and no protocol was prepared.

## Appendix A

Search strategies

Pubmed:

((“Randomised Controlled Trials as Topic”[Mesh] OR “Randomised controlled trial*”[tw] OR “randomised controlled trial*”[tw] OR “RCT”[tw] OR “randomised trial*”[tw] OR “randomised trial*”[tw] OR “randomised control* trial*”[tw] OR “randomised control* trial*”[tw]) OR (“clinical trials as topic”[mesh] OR “clinical trial*”[tw] OR “non-clinical trial*”[tw] OR “non clinical trial*”[tw])OR (“Non-Randomised Controlled Trials as Topic”[Mesh] OR “non-randomised trial*”[tw] OR “non-randomised trial*”[tw]) OR (“Controlled Before-After Studies”[Mesh] OR “controlled before-after stud*”[tw] OR “controlled before and after stud*”[tw] OR “controlled before & after stud*”[tw] OR “controlled before-and-after stud*”[tw]) OR (“Interrupted Time Series Analysis”[Mesh] OR “interrupted time series”[tw] OR “time series”[tw] OR “interrupted time series stud*”[tw] OR “time series stud*”[tw]) OR (“repeated measures”[tw] OR “repeated measures stud*”[tw])) AND (“Family Practice”[Mesh] OR “General Practice”[Mesh] OR “general practitioners”[mesh] OR “Primary Health Care”[mesh] OR “Physicians, Primary Care”[tw] OR (“primary”[tw] AND (“care”[tw] OR “healthcare”[tw] OR “health care”[tw])) OR “primary care provider*”[tw] OR “primary care physician*”[tw] OR “family medicine”[tw] OR “family practice”[tw] OR “general practitioner*”[tw] OR “general practice*”[tw] OR “general practitioner*”[tw] OR “family doctor*”[tw] OR “general doctor*”[tw]) OR (“Ambulatory Care”[Mesh] OR “Outpatients”[Mesh] OR “ambulatory care”[tw] OR “outpatient care”[tw] OR “outpatient*”[tw]) AND (“Learning Health System”[Mesh] OR “Educational Technology”[Mesh] OR “Academic Performance”[Mesh] OR “Reminder Systems”[Mesh] OR “Quality Assurance, Health Care”[Mesh] OR “Education, Medical, Continuing”[Mesh] OR (“audit”[tw] AND “feedback”[tw]) OR “community of practice”[tw] OR “communities of practice”[tw] OR “educational game*”[tw] OR “educational meeting*”[tw] OR “educational material*”[tw] OR “educational outreach visit*”[tw] OR “academic detailing”[tw] OR “inter-professional education”[tw] OR “interprofessional education”[tw] OR “local consensus process*”[tw] OR “local opinion leader*”[tw] OR “patient-mediated intervention*”[tw] OR “patient mediated intervention*”[tw] OR “reminder”[tw] OR “tailored intervention*”[tw] OR “financial incentive*”[tw] OR “intervention*”[tw] OR “program*”[tw] OR “algorithms”[mesh] OR “algorithm*”[tw] OR “one-on-one session*”[tw] OR “one on one session*”[tw]) AND ((“Anti-Bacterial Agents”[MesH] OR ((“antibiotic*”[tw] OR “antimicrobial*”[tw] OR “anti-microbial*”[tw] OR “antibacterial*”[tw] OR “anti-bacterial*”[tw] OR “UTI”[tw]) AND (“therap*”[tw] OR “treatments*”[tw] OR “management”[tw] OR “drug*”[tw] OR “agent*”[tw]))) AND (((“appropriate*”[tw] OR “inappropriate*”[tw] OR “unnecessary*”[tw] OR “necessary*”[tw] OR “acceptibl*”[tw] OR “unacceptabl*”[tw] OR “improv*”[tw] OR “rate*”[tw]) AND (“prescri*”[tw] OR “recipe*”[tw] OR “dispens*”[tw])) OR “Guideline Adherence”[Mesh] OR ((“guideline*”[tw] OR “protocol*”[tw] OR “instruction*”[tw]) AND (“adher*”[tw] OR “non-adher*”[tw] OR “nonadher*”[tw] OR “accord*”[tw] OR “non-accord*”[tw] OR “nonaccord*”[tw] OR “concord*”[tw] OR “non-concord*”[tw])))) AND (“Urinary Tract Infections”[mesh] OR “urinary tract infection*”[tw] OR “cystitis”[tw] OR “UTI”[tw] OR “UTIs”[tw] OR “bacteriuria”[mesh] OR “bacteriuria”[tw] OR “pyuria”[mesh] OR “pyuria”[tw])

Cochrane Central Register of Controlled Trials:

ID	Search	Hits
#1	MeSH descriptor: [Randomised Controlled Trials as Topic] explode all trees	15180
#2	MeSH descriptor: [Non-Randomised Controlled Trials as Topic] explode all trees	88
#3	MeSH descriptor: [Controlled Before-After Studies] explode all trees	85
#4	MeSH descriptor: [Interrupted Time Series Analysis] explode all trees	67
#5	random* control* trial* OR RCT OR random* trial OR non-random* trial OR controlled before-after stud* OR interrupted time serie* OR interrupted time serie* stud* OR repeated measure* OR repeated measures stud*	1327639
#6	(#1 OR #2 OR #3 OR #4 OR #5)	1327654
#7	MeSH descriptor: [Family Practice] explode all trees	1981
#8	MeSH descriptor: [General Practice] explode all trees	2504
#9	MeSH descriptor: [General Practitioners] explode all trees	336
#10	MeSH descriptor: [Primary Health Care] explode all trees	8364
#11	MeSH descriptor: [Ambulatory Care] explode all trees	3753
#12	MeSH descriptor: [Outpatients] explode all trees	1379
#13	(primary AND (care OR health care OR healthcare)) OR primary care provider* OR primary care physician* OR family medicine OR family practice OR general practitioner* OR family doctor* general practice OR ambulatory care OR outpatient OR outpatient care	163256
#14	(#7 OR #8 OR #9 OR #10 OR #11 OR #12 OR #13)	166331
#15	(audit AND feedback) OR communit* of practice OR educational game* OR educational meeting* OR educational meeting* educational material* OR educational outreach visit* OR academic detailing OR inter-professional education OR interprofessional education OR local consensus process* OR local opinion leader* OR patient-mediated intervention* OR patientmediated intervention* OR patient mediated intervention* OR reminder* OR delayed prescription* OR delayed antibiotic prescription* OR tailored intervention* OR financial incentive* OR intervention* OR program*	555221
#16	MeSH descriptor: [Learning Health System] explode all trees	2
#17	MeSH descriptor: [Educational Technology] explode all trees	3991
#18	MeSH descriptor: [Academic Performance] explode all trees	116
#19	MeSH descriptor: [Reminder Systems] explode all trees	1016
#20	MeSH descriptor: [Quality Assurance, Health Care] explode all trees	3426
#21	MeSH descriptor: [Education, Medical, Continuing] explode all trees	747
#22	(#15 OR #17 OR #18 OR #19 OR #20 OR #21)	558815
#23	MeSH descriptor: [Anti-Bacterial Agents] explode all trees	13002
#24	antibiotic* OR antimicrobial* OR anti-microbial* OR antibacterial* OR anti-bacterial*	49434
#25	(#23 OR #24)	50346
#26	(prescr* OR recipe* OR course* OR dispens*)	120534
#27	(#25 AND #26)	8598
#28	MeSH descriptor: [Urinary Tract Infections] explode all trees	2645
#29	MeSH descriptor: [Cystitis] explode all trees	470
#30	urinary tract infection* OR UTI OR UTIs OR cystitis OR pyelonephritis OR prostatitis	13518
#31	(#28 OR #29 OR #30)	13765
#32	#6 AND #14 AND #22 AND #27 AND #31	331

Web of Science:

#1	TS = (random* control* trial* OR random* trial* OR clinical trial* OR non-clinical trial* OR non clinical trial* OR non-random* trial OR non-random* trial OR controlled before-after stud* OR controlled before & after stud* OR controlled before and after stud* OR interrupted time serie* OR time serie* OR repeated measure* OR repeated measure* stud*)	1903206
#2	TS = (family practice* OR general practice* OR general practitioner* OR primary health care OR primary care physician* OR (primary AND (care OR health care OR healthcare)) OR primary care provider* OR family medicine OR family practice OR general practitioner* OR family doctor* OR ambulatory care OR outpatient*)	777538
#3	TS = ((audit AND feedback) OR communit* of practice OR learning health system* OR educational technology* OR academic performance* OR reminder system* OR quality assurance* OR continuing medical education OR educational game* OR educational meeting* OR educational material* OR educational outreach visit* OR academic detailing OR inter-professional education OR interprofessional education OR local consensus process* OR local opinion leader* OR patient-mediated intervention* OR patient mediated intervention* OR reminder* OR tailored intervention* OR financial incentive* OR intervention* OR program* OR algorithm* OR one-on-one session* OR one on one session*)	5774938
#4	TS = ((antibiotic* OR antimicrobial* OR anti-microbial* OR antibacterial* OR anti-bacterial*) AND (prescri* OR recipe* OR course* OR dispens*))	40255
#5	TS = (urinary tract infection* OR UTI OR UTIs OR cystitis OR bacteriuria OR pyuria OR pyelonephritis OR prostatitis)	69020
#6	#1 AND #2 AND #3 AND #4 AND #5	106

## Appendix B

**Table A1:** Intervention taxonomy of the Cochrane Collaboration Effective Practice and Organization of Care (EPOC) [39].

Intervention	Definition
Audit & feedback	A summary of health workers’ performance over a specified period of time, given to them in a written, electronic, or verbal format. The summary may include recommendations for clinical action.
Communities of practice	Groups of people with a common interest who deepen their knowledge and expertise in this area by interacting on an ongoing basis.
Educational games	The use of games as an educational strategy to improve standards of care.
Educational materials	Distribution to individuals, or groups, of educational materials to support clinical care, i.e., any intervention in which knowledge is distributed. For example, this may be facilitated by the internet, learning critical appraisal skills; skills for electronic retrieval of information, diagnostic formulation; question formulation.
Educational meetings	Courses, workshops, conferences, or other educational meetings.
Educational outreaching, academic detailing	Personal visits by a trained person to health workers in their own settings, to provide information with the aim of changing practice.
Interprofessional education	Continuing education for health professionals that involves more than one profession in joint, interactive learning.
Local consensus processes	Formal or informal local consensus processes, for example agreeing a clinical protocol to manage a patient group, adapting a guideline for a local health system, or promoting the implementation of guidelines.
Local opinion leaders	The identification and use of identifiable local opinion leaders to promote good clinical practice.
Patient-mediated interventions	Any intervention aimed at changing the performance of healthcare professionals through interactions with patients, or information provided by or to patients.
Reminders	Manual or computerized interventions that prompt health workers to perform an action during a consultation with a patient, for example computer decision support systems.
Tailored interventions	Interventions to change practice that are selected based on an assessment of barriers to change, for example through interviews or surveys.
